# Identifying dietary consumption patterns from survey data: a Bayesian nonparametric latent class model

**DOI:** 10.1093/jrsssa/qnad135

**Published:** 2023-12-12

**Authors:** Briana J K Stephenson, Stephanie M Wu, Francesca Dominici

**Affiliations:** Department of Biostatistics, Harvard T.H. Chan School of Public Health, Boston, MA, USA; Department of Biostatistics, Harvard T.H. Chan School of Public Health, Boston, MA, USA; Department of Biostatistics, Harvard T.H. Chan School of Public Health, Boston, MA, USA

**Keywords:** Bayesian nonparametrics, dietary patterns, latent class model, NHANES, survey design

## Abstract

Dietary assessments provide the snapshots of population-based dietary habits. Questions remain about how generalisable those snapshots are in national survey data, where certain subgroups are sampled disproportionately. We propose a Bayesian overfitted latent class model to derive dietary patterns, accounting for survey design and sampling variability. Compared to standard approaches, our model showed improved identifiability of the true population pattern and prevalence in simulation. We focus application of this model to identify the intake patterns of adults living at or below the 130% poverty income level. Five dietary patterns were identified and characterised by reproducible code/data made available to encourage further research.

## 1 Introduction

### 1.1 Motivation

The impact of poor diet has continually devastated the U.S., accounting for over 500,000 deaths annually, with 84% of those deaths due to cardiovascular disease (CVD) (Mokdad et al., 2018; Roth et al., 2018). The negative health impacts of poor diet disproportionately affect low-income and racial minority populations (Brown et al., 2018; Fahlman et al., 2010). Understanding the dietary consumption behaviours that contribute to poor health in these target populations may help in tailoring interventions and resources to improve their nutritional health.

Through the implementation of complex survey designs and targeted recruitment strategies, researchers are able to obtain more representative population samples in an effort to better understand populations of greatest interest. Consequently, survey sampling methodologies have been developed to improve population-based estimates and generate appropriate inference based on the sampled data.

While low-income and racial minority adults are a population at greatest risk of poor diet and subsequently poorer health outcomes, they are often underrepresented in the survey studies (Tourangeau et al., 2014). In an effort to achieve a more nationally representative sample, surveys such as the National Health and Nutrition Examination Survey (NHANES) have corrected for this underrepresentation by oversampling demographics of greater public health interest to improve the accuracy and reliability of national-based estimates of health outcomes and exposures (Zipf et al., 2013). Unfortunately, most of the current statistical approaches for deriving dietary patterns from survey data do not incorporate the weights during estimation, which could lead to biased and inconsistent data-driven patterns for population demographics.

Latent class models (LCM) are an effective tool to comprehensively analyse the consumption patterns of a full set of foods included on a diet assessment (Keshteli et al., 2015; Sotres-Alvarez et al., 2010). Implementation of this procedure is available on commonly used statistical software such as SAS (Proc LCA) and R (poLCA) and offer parameter estimation of latent class model parameters via frequentist algorithms (e.g. expectation-maximisation and Newton–Raphson) (Lanza et al., 2007; Linzer & Lewis, 2011; B. Muthén & Shedden, 1999). Bayesian estimation is accomplished through an R package (BayesLCA), but is limited to binary consumption responses (White & Murphy, 2014).

Patterns derived from LCA are dependent on the observed study data. This is of concern when the study data are not representative of the study population. For example, historically, certain subgroups of the population have been undersampled and underrepresented in studies. In other scenarios, surveys may purposely oversample subgroups to gain more information from them. Demographics that dominate in a population often mask dietary habits of smaller-sized demographics, which may deviate from the majority habit. Study designs have strived to correct for this through the implementation of sampling weights that account for underrepresentation and nonresponse. However, none of these standard packages previously described incorporate sampling weights directly into the estimation procedures. Mplus is one of the few statistical software available to adjust for complex survey design, but is limited under a frequentist framework, which can present issues with matrix inversion and computational demand when handling the high dimensionality of diet data, which can be large and sparse for rarely consumed food items (L. K. Muthén & Muthén, 2017).

### 1.2 Potential solutions in Bayesian nonparametrics

Bayesian nonparametrics offers a more efficient solution that is able to (1) accommodate the complex high dimensionality of dietary intake data, (2) handle large-sized populations, such as the U.S., (3) reduce multiple model testing and fitting to determine the appropriate number of patterns, (4) preserve model stability in the presence of sparsely consumed foods, and (5) integrate prior information with observed data. These features improve parameter estimation and subsequent population inference (Hjort et al., 2010; J. S. Liu, 2008) and also provide advantages in computational feasibility and flexibility of pattern dimensionality (Bartolucci et al., 2017).

Bayesian survey data applications have centred mostly on generating inference for derived population-based estimates (Gunawan et al., 2020; Savitsky & Toth, 2016; Si et al., 2015), but have not been fully explored in regard to model-based clustering. Bayesian nonparametric mixture models that utilised dietary intake data either did not contain complex survey data (De Vito et al., 2019; Fahey et al., 2007; Stephenson, Herring, et al., 2020), or applied sampling weights posthoc after model parameter estimation was complete (De Vito et al., 2022; Stephenson, Sotres-Alvarez, et al., 2020). Kunihama et al. (2016) is one of the few who introduced a sampling algorithm that incorporates survey weights directly into the estimation, but did not take into account sampling variability present in nationally representative surveys.

Our overall objective is to examine the dietary patterns of low-income adults in the U.S. This adult subpopulation represents a minority of the US and the patterns of this demographic are often masked by the majority of adults not classified as low-income. We have built upon the survey sampling framework and added the following contributions: (1) implemented an overfitted latent class model, which is asymptotically similar to the Dirichlet Process mixture model; (2) extended and integrated the works of Kunihama et al. (2016) and Savitsky and Toth (2016) to generate population-based estimates that also adjust for sampling variability in the survey design; (3) demonstrated the utility of this approach by applying this model to publicly available national survey data to derive nationally representative dietary consumption patterns of low-income adults in the U.S. from 2011 to 2018; and (4) provided publicly available reproducible code for researchers to apply this technique on future national dietary survey data.

We organise this paper as follows: Section 2 describes our proposed weighted overfitted latent class model (wtOLCM). Section 3 compares our model with current model-based approaches for survey data via a simulation study. Section 4 describes the National Health and Nutrition Examination Survey. Section 5 presents results of the method applied to the National Health and Nutrition Examination Survey.

## 2 Weighted overfitted latent class model

A weighted overfitted latent class model is a Bayesian nonparametric technique that can be used to identify subgroups or clusters within a survey sample that share common behaviours among a set of observed nominal variables (Van Havre et al., 2015). The model assumes that each individual has a known sampling weight. These sampling weights are integrated into the likelihood to form a pseudo-likelihood that mimics the likelihood relative to the size of the target population, followed by the generation of pseudo-posterior inference. It can be seen as an extension of the latent class model, which typically requires multiple fits and post-hoc testing to determine the appropriate number of latent classes or patterns. This is of great benefit since many of the information criterion measures differ in penalisation and therefore evaluate model fit differently, yielding inconsistent results (Grimm et al., 2021; C. Kim, 2021; Xue et al., 2017). The overfitted latent class model removes this redundancy by overfitting the model with a large number of latent classes (or clusters) and allowing a data-driven approach to choosing the number of latent clusters. Empty clusters are able to drop out of the model during the Markov chain Monte Carlo Gibbs sampling algorithm, and nonempty clusters remain. Each participant is assigned to one of the derived clusters, corresponding to a dietary pattern. The overfitted structure is also asymptotically equivalent to the Dirichlet Process model, allowing additional flexibility within a Bayesian nonparametric framework (Van Havre et al., 2015).

### 2.1 OLCM

We define some notation of the standard latent class model, with a sampled population of size *n* and *K* unique dietary patterns, where each pattern describes the consumption of *p* food items. Let $y_{i\cdot }=(y_{i1},\ldots ,y_{ip})$ denote the set of *p* observed food items. Each observed food item, $y_{ij}$, is categorical, where $y_{ij}\in \{1,2,\ldots ,d_{j}\}$ is individuals *i*’s consumption level for food item *j*. Let $\pi_{k}$ denote the probability of assignment to dietary pattern k∈{1,…,K}, and $\pi =(\pi_{1},\ldots ,\pi_{K})$. The dietary pattern assignment of individual i∈{1,…,n} from the sampled population is denoted by $z_{i}$. Let $\theta_{jc|k}$ denote the probability of consuming food item *j*, at the $c\in \{1,\ldots ,d_{j}\}$ consumption level, given an individual’s assignment to diet pattern *k*, where $d_{j}$ is the maximum consumption level for food item *j*, and $\theta =\{\theta_{jc|k}{\}}_{j,k}$. The subject-specific likelihood is then defined as


(1) $$Pr(y_{i\cdot }|\theta ,\pi ,z_{i})=\sum_{k=1}^{K}\pi_{k}\prod_{j=1}^{p}\prod_{c=1}^{d_{j}}\theta_{jc|k}^{1(y_{ij}=c|z_{i}=k)}.$$

The likelihood for the overfitted latent class model shares the same structure as that of the standard latent class model shown in (1), but since *K* is typically not known in practice, it is fixed to an exceedingly large number that asymptotically simulates an infinite mixture model (Van Havre et al., 2015). Under a Bayesian estimation framework, the model parameters are updated in the Gibbs sampler based on the number of observed individuals classified to a given latent class or consumption level. For example, exploiting the convenience of conjugacy, the probability vector, $\pi =(\pi_{1},\ldots ,\pi_{K})$, follows a Dirichlet prior and conditional posterior with hyperparameters for each latent class defined as $\left(\alpha_{1},\ldots ,\alpha_{K}\right)$:


(2) $$\begin{array}{rl} \pi =(\pi_{1},\ldots ,\pi_{K}) & ∼Dir(\alpha_{1},\ldots ,\alpha_{K})\\ \left(\pi_{1},\ldots ,\pi_{K}|y_{\cdot \cdot },z_{\cdot }\right) & ∼Dir\left(\alpha_{1}+\sum_{i=1}^{n}1(z_{i}=1),\ldots ,\alpha_{K}+\sum_{i=1}^{n}1(z_{i}=K)\right), \end{array}$$

where $y_{\cdot \cdot }=(y_{1\cdot },\ldots ,y_{n\cdot })$ and $z_{\cdot }=(z_{1},\ldots ,z_{n})$.

With no prior knowledge on the number of classes, we utilise a noninformative, flat Dirichlet prior, where $\alpha_{1}=\alpha_{2}=\cdots =\alpha_{K}=\alpha$. A flat Dirichlet prior allows for an equal weighting of confidence over cluster size parity. In other words, by setting α=1, we assume the probability of assignment to any given latent class is equal. If we had more information on either the number of latent classes or the size of the respective latent classes, we would define more informative hyperparameter values into the prior distribution. Larger $\alpha_{k}$ values can be given to latent classes that are expected to exist and have a substantial relative size or weight. Smaller hyperparameter values can be given to latent classes that are either not expected to exist or relatively smaller in size. For example, if a sampled dataset is expected to have only three latent classes, where the first latent class is twice as large as the other two, one may define the hyperparameters, such that $\left(\alpha_{1}=2,\alpha_{2}=1,\alpha_{3}=1,\alpha_{4}=0.01,\ldots ,\alpha_{K}=0.01\right)$. The magnitude of *α* also moderates the rate of growth for nonempty latent classes. Smaller hyperparameter values also indicate a slower rate at which nonempty latent classes will form. So, in the previous example, the hyperparameter setup indicates that the first three latent classes will form much faster than any subsequent latent classes, unless the data overwhelmingly favour that decision.

Similarly, the lack of prior knowledge assumption can be translated also to the consumption pattern, or latent class distribution, of each observed food. In this case, the different consumption levels will all have nonzero probability values to have a full set of nonempty levels, as opposed to the near-zero probability values used for $\pi_{h}$ which allowed empty clusters to remain. Therefore, the flat, Dirichlet prior distribution could have a hyperparameter value of 1 to initialise the different consumption level probabilities to equal weights, $\left\{\theta_{j\cdot |k}=(\theta_{j1|k},\ldots ,\theta_{jd_{j}|k}){\}}_{j=1}^{p}∼Dir(\gamma_{1},\ldots ,\gamma_{d_{j}}\right)$ for all k∈{1,2,…,K}, where $\gamma_{1}=\gamma_{2}=\gamma_{d_{j}}=\gamma =1$.

### 2.2 Extension to wtOLCM

In a typical survey setting, individuals are assigned sampling weights, which may describe the inclusion probability, nonresponse, or calibration weights. Incorporating these weights in a Bayesian setting serves as a natural extension to the overfitted latent class model. As described by Kunihama et al. (2016), information used to update each model parameter is enhanced with weights, simulating a pseudo-like population that is similar in size and structure to the target population. A normalisation constant is used to ensure the weights sum to the target population. This enables dietary patterns to form in accordance with the target population, but does not consider changes that can occur in size and composition from one sampled population to another. However, sampling variability should be considered in the model, and precision estimates should reflect the sample size rather than the population size. Otherwise, uncertainty surrounding model estimation will be biased. To address this limitation, we instead propose to normalise the sampling weights to sum to the sample size as proposed by Bartolucci et al. (2016) and Savitsky and Toth (2016). This will account for sampling variability while allowing model estimates to generalise better to the target population.

Let $w_{i}$ denote the sampling weight of study participant i∈{1,…,n}. We impose a fixed normalisation constant, *κ*, where $\kappa =\frac{\sum_{i}w_{i}}{n}$, with *n* denoting the study sample size. With this newly defined *κ* and with $w_{\cdot }=(w_{1},\ldots ,w_{n})$, the conditional posterior of the probability of assignment vector, $\pi =(\pi_{1},\ldots ,\pi_{K})$, updates based on the weighted number of participants assigned to each pattern


(3) $$(\pi_{1},\ldots ,\pi_{K}|y_{\cdot \cdot },z_{\cdot },w_{\cdot })∼Dir\left(\alpha_{1}+\frac{1}{\kappa }\sum_{i=1}^{n}w_{i}\times 1(z_{i}=1),\ldots ,\alpha_{K}+\frac{1}{\kappa }\sum_{i=1}^{n}w_{i}\times 1(z_{i}=K)\right).$$

Similarly, for the consumption level distribution of each dietary pattern, $f(y_{i\cdot }|z_{i}=k)=$  $\prod_{j=1}^{p}\prod_{c=1}^{d_{j}}\theta_{jc|k}^{1(y_{ij}=c|z_{i}=k)}$, k∈{1,…,K}, updates for the conditional posteriors of the probabilities of consumption are based on the weighted number of participants that share dietary consumption behaviours. For all j∈{1,…,p} and k∈{1,…,K}


(4) $$\theta_{j\cdot |k}∼Dir\left(\gamma +\frac{1}{\kappa }\sum_{i:z_{i}=k}w_{i}\times 1(y_{ij}=1),\ldots ,\gamma +\frac{1}{\kappa }\sum_{i:z_{i}=k}w_{i}\times 1(y_{ij}=d_{j})\right).$$

The full likelihood model is written as


(5) $$f(y|\pi ,\theta ,z_{i})=\prod_{i=1}^{n}{\left\{\sum_{k=1}^{K}\pi_{k}\prod_{j=1}^{p}\prod_{c=1}^{d}\theta_{jc|k}^{1(y_{ij}=c|z_{i}=k)}\right\}}^{\frac{{\tilde{w}}_{i}}{\kappa }}$$

### 2.3 Prior and posterior considerations

The overfitted finite mixture model framework used for the wtOLCM requires some additional considerations including sensitivities to prior selection, label switching, and latent class identification. All Bayesian mixture models are prone to sensitivity to the prior selected. As highlighted in Section 2.1, the number of latent classes in the model can vary based on the hyperparameters defined in the prior distribution. Typically, the number of clusters grows logarithmically with the sample size. Extending to a survey setting, where the data have been augmented to a pseudo-population, similar in size to the target population, there can be even more of an increase in the number of latent classes that will appear. Yet, several of these additional clusters may be redundancies, weakly separated, or nonexistent in truth (Miller & Harrison, 2018). The decision of prior selection should be appropriately determined with priority given to the scientific context and interpretability. Sensitivity analysis can be performed to compare model results based on different prior implementations, such as a parallel tempered prior or an asymmetric Dirichlet prior, and consistency across pattern identification and interpretability of results can be evaluated. The priors implemented in this study for simulation and application, prioritised a modest rate of cluster growth to permit enough separation of patterns and preserve diet interpretability.

Label switching is a problem prevalent in all mixture models. It occurs when the labels of latent classes are swapped randomly in the MCMC, but due to exchangeability, the likelihood remains invariant throughout (Stephens, 2000). This greatly impacts posterior estimation and inference of the respective distributions of each latent class because attributes describing a single latent class in one iteration will be summarised with attributes describing a different cluster in a subsequent iteration, when generating summaries from the posterior distribution. This decreases interpretability of the true size of each latent class and its respective characterised distribution. Since information on the relative sizes of each latent class or their distribution is not known prior to analysis, there is no way to follow or tag a given cluster as it moves throughout the sampling algorithm. Several solutions have been proposed in how to handle this phenomenon (Rodríguez & Walker, 2014; Sperrin et al., 2010; Stephens, 2000). We resolve this using approaches introduced by Krebs (1989) and Medvedovic and Sivaganesan (2002) where individuals who tend to cluster together are followed throughout the MCMC and retain the respective labels at each iteration. This is achieved in postprocessing with a hierarchical clustering on a similarity matrix of size n×n using complete linkage approach. Matrix elements contain pairwise posterior probabilities of two subjects being clustered together in each MCMC iteration. The latent class assignment is then determined through a reordering step where individuals allocated to the derived number of nonempty latent classes from the posterior are ordered based on the label in which they fell for that iteration.

The derived number of nonempty latent classes is seldom known a priori. Standard latent class models determine this number based on multiple model fittings and comparison of best-fit information criterion measures (e.g. Watanabe-Akaike Information Criterion, Average Extended Bayesian Information Criterion, Deviance Information Criterion, Bayesian Information Criterion). However, each of these criterions implement different penalties based on complexity and model estimation, yielding inconsistent results across different measures. This limitation is what makes the overfitted mixture model framework so attractive. Nonexistent clusters are expected to remain empty in the algorithm, leaving behind the true number of nonempty latent classes. Yet, inconsistencies in the number of nonempty clusters can still persist, resulting in the appearance of redundant and small, nonexistent clusters (Miller & Harrison, 2018). While redundant cluster patterns are easy to identify and merge in postprocessing of multivariate categorical data, the sparsity issue of removing small or empty classes, requires implementing a threshold to better define cluster existence. Prior simulation studies with similar data applications have proposed that a suitable threshold to define a nonempty existing latent class is one which contains at least 5% of the sampled participants (Stephenson, Herring, et al., 2020; Stephenson, Sotres-Alvarez, et al., 2020; Stephenson & Willett, 2023). This threshold can be implemented in resolving the relabelling and reordering step of latent class assignment during postprocessing, allocating individuals into one of the derived latent classes that meets the minimum threshold.

## 3 Simulation study

### 3.1 Survey-weighted approaches

We performed our simulation study under three different approaches for handling survey data. Method 1 serves as our control, a standard overfitted latent class model, where sample weights are ignored (unweighted OLCM). Method 2 provides an alternative approach, a weighted finite population Bayesian bootstrap (WFPBB) (Dong et al., 2014; Gunawan et al., 2020), where pseudo-representative samples are generated by using survey weights to ‘undo’ the unequal sampling scheme and impute synthetic populations, and then approximate simple random samples are drawn from these synthetic populations. This application of the WFPBB method builds on earlier work of pseudo-population generation through multiple imputation techniques (Raghunathan et al., 2003; Zhou et al., 2016). Implementation details of this method are provided in online supplementary material Section 1. Method 3 is our proposed wtOLCM that extends the work of Kunihama et al. (2016) and Savitsky and Toth (2016) where the sample weights are directly incorporated into the sampling algorithm, as detailed in Section 2. Our simulation study will evaluate how well these three methods are able to identify the true population prevalence of dietary patterns using the sampled data.

### 3.2 Simulation setup

The goal of our simulation study is to compare how well the three methods are able to identify the true population prevalence, as well as the composition of the true population patterns. Algorithm run time is also compared for computational reference. We consider a simulated population of size N=5,000. A total of $K_{true}=3$ patterns exist in the population with probability distribution $\pi_{true}=(0.1,0.3,0.6)$. Each pattern consists of p=50 categorical variables that can take on values 1, 2, 3, or 4. For case A, the mode was set at 0.85 for the true pattern value of interest, and at 0.05 for the remaining three values. To evaluate under additional noise, case B was performed where the mode was set at 0.55, and 0.15 for all other remaining values. Pattern 1 was defined with a mode at level 3 for the first 25 variables, and a mode at level 1 for the remaining 25 variables. Pattern 2 was defined with a mode at level 2 for the first 10 variables, and a mode at level 4 for the remaining 40 variables. Pattern 3 was defined with a mode at level 1 for the first 10 variables, a mode at level 2 for the next 20 variables, and a mode at level 3 for the remaining 20 variables. Subjects were initially assigned to one of these three patterns, and the subject-specific observed data were simulated by drawing from a multinomial distribution for each of the 50 corresponding variables described above based on the assigned pattern. The total population was comprised of S=4 disproportionate subpopulations containing varied distributions of the three patterns (online supplementary material Table 1). A subset of 100 subjects were randomly selected from each of the simulated subpopulations, totalling n=400 simulated subjects in each sample dataset. A total of 100 simulated datasets were generated for replicability under an overfitted model of K=50 clusters under the three previously described approaches in Section 3.1.

Assuming no prior knowledge on the number of true clusters and the relative distribution of each variable’s categorical variables for all models, we utilised a flat Dirichlet prior with $\alpha =\frac{1}{K}=\frac{1}{50}$ for $\pi_{\cdot }$ to conservatively moderate the rate of cluster growth as suggested by Rousseau and Mengersen (2011). Each latent class pattern distribution was also fit with a noninformative, flat Dirichlet prior with γ=1 for all j∈(1,…,p) to initialise an equal weight distribution among all categorical variables. All analysis was performed using MATLAB 2021a with an MCMC of 25,000 iterations and a burn-in of 15,000 and thinning every five iterations.

### 3.3 Simulation results

**Figure 1.**
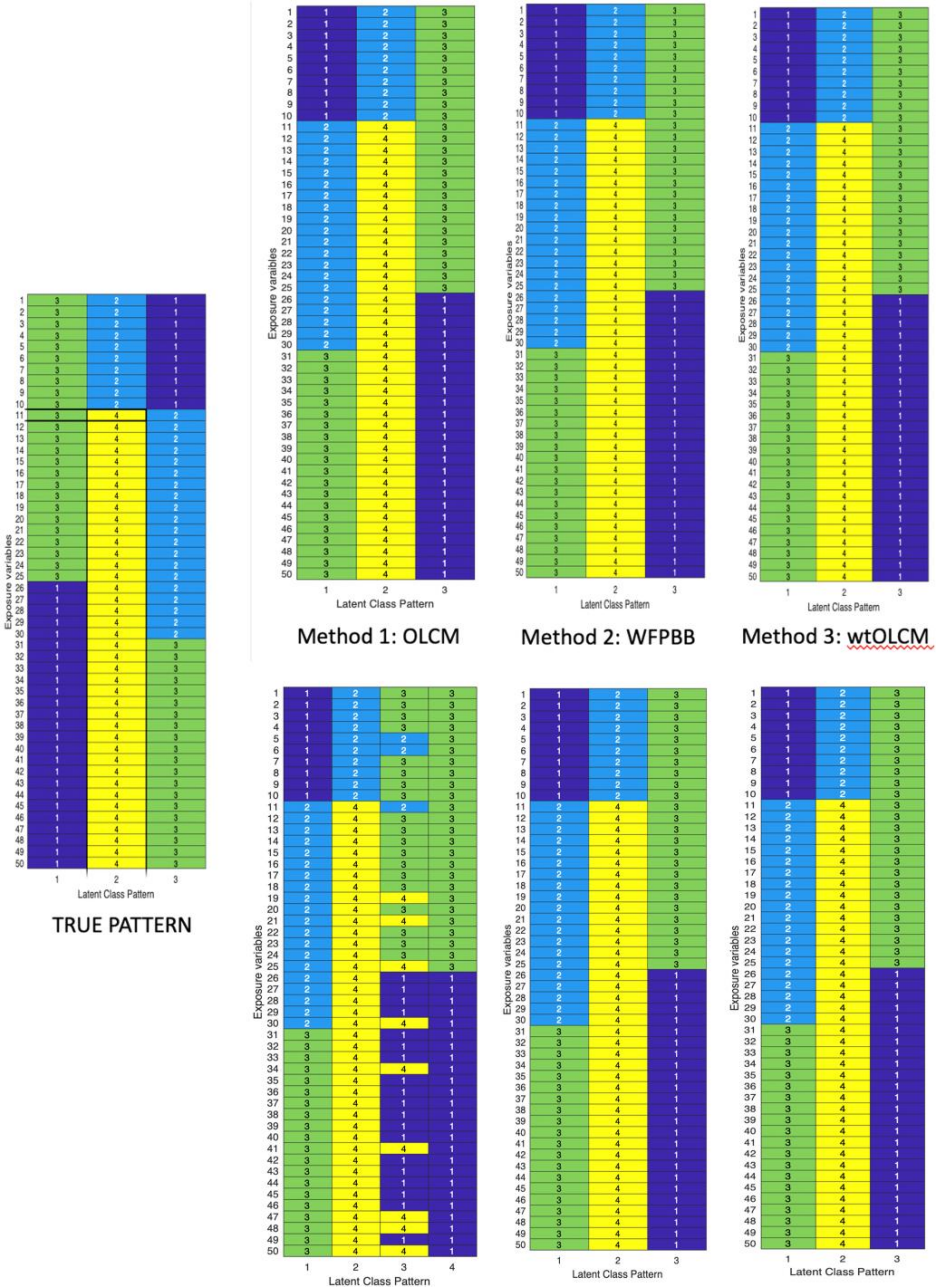
Modal consumption patterns identified from respective models compared to truth. Method 1: overfitted latent class model, ignoring weights; Method 2: weighted finite population Bayesian bootstrap; Method 3: weighted overfitted latent class model. Top indicates pattern under simulation case A. Bottom indicates pattern under simulation B. The additional noisy cluster is illustrated in Method 1, where the size of this pattern had a prevalence of 0.02.

Model diagnostics indicated good mixing and successful convergence of model parameters across all three methods. Derived patterns were identified by setting the modal response to be the categorical level of each exposure variable that had the highest posterior probability of consumption. As illustrated in Figure 1, each of the methods successfully identified the true number of patterns (K=3) as well as the modal response patterns in case A. The additional noise incorporated in case B generated additional clusters containing redundancies to the true patterns when using the unweighted method, but these were small in size $\left(\pi_{4}<0.03\right)$.

**Figure 2.**
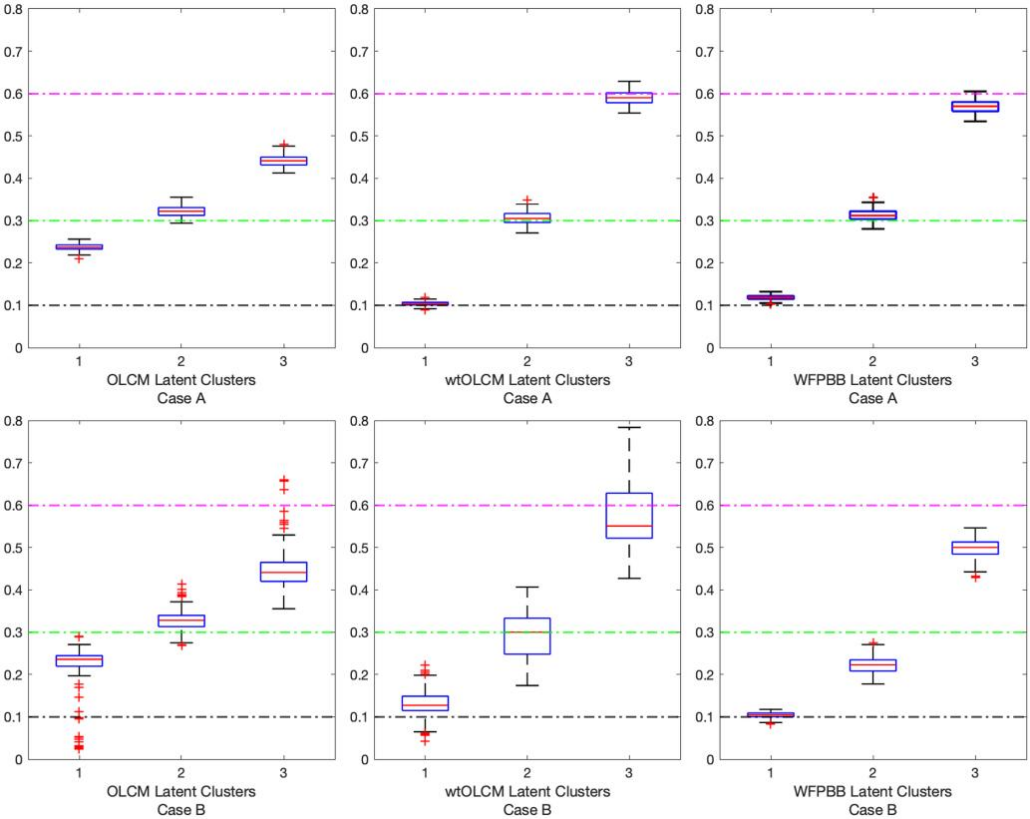
Predicted population prevalence from unweighted and weighted estimation approaches. Expected prevalence for each respective cluster is 0.1 (lowest reference line), 0.3 (middle reference line), and 0.6 (highest reference line).

Bias and precision in the expected prevalence of the patterns did differ across the three methods, as illustrated in Figure 2. The prevalence estimates for the unweighted method are clearly biased, whereas the estimates of the wtOLCM method show the least amount of bias, with slight sensitivity to noise for case B. Under the unweighted method, the MSE of the true pattern prevalence in the population was 0.015 and 0.016, respectively. Both the WFPBB and the wtOLCM methods had an improved estimation of the population prevalence compared to the unweighted case. Among all methods, wtOLCM had the best coverage of the true population prevalence [$\mathrm{MSE}({\hat{\pi }}_{A})=1.3\times 10^{-4},\mathrm{MSE}({\hat{\pi }}_{B})=0.002$] in both simulation cases.

## 4 NHANES

The NHANES is a population-based survey designed to assess the health and nutritional status of adults and children in the U.S. The survey samples at least 9,000 people across various socio-economic status levels each year residing in 15 randomly selected counties in the U.S. Starting in 2011, NHANES created more granularity to the race/ethnicity variable, separating Mexican–American from Other Hispanic participants, as well as adding an identifier for Non-Hispanic Asian. For the scope of this study, we limited analysis to survey cycles containing the seven race/ethnicity groups, and adults aged 20 and over. Low-income participants were identified as those reporting at or below the 130% poverty income level.

Dietary intake was collected via the ‘What We Eat in America’ survey component of NHANES. Food items and beverages were consumed and recorded via two 24-hour recalls. Nutrients comprising these reported food/beverage items were calculated using the Food and Nutrition Database for Dietary Studies and then converted into food pattern equivalents per 100 g of consumption based on the Dietary Guidelines for Americans (Bowman et al., 2017, 2016, 2018; Committee et al., 2015).

Dietary consumption data were summarised as 29 food groups and pooled across four NHANES survey cycles: 2011–2012, 2013–2014, 2015–2016, and 2017–2018. Consumption levels were derived by segmenting the data into no consumption (none=0%) and tertiles of positive consumption (L. Liu et al., 2019; Sotres-Alvarez et al., 2013). NHANES dietary weights were adjusted for the pooled survey years in accordance with protocols outlined in NHANES analytic guidelines (T.-C. Chen et al., 2020; National Center for Health Statistics and Surveys, 2018).

Demographic information of the low-income adult participants collected in NHANES is detailed in online supplementary material Table 2. The low-income sampled population reflected a demographic with the larger proportion of participants identifying as non-Hispanic White (47.6%), female (54.5%), and between 20 and 34 years old (35.7%). This sampled population reported an alternative healthy eating index (AHEI-2015) score of 49.2 out of 100, which is less than the overall national average of 58 out of 100. The mean Framingham 10-year risk score indicated a low risk of a CVD outcome occurring in the next 10 years (FRS=7.7).

### 4.1 Fitting NHANES to weighted overfitted latent class model

For our model, the normalisation constant $\left(\kappa =9.79\times 10^{3}\right)$ was calculated based on the sum of the sampled weights divided by the total sample size (n=7,561). We overfit the model with K=50 latent classes. Estimation was performed using a Gibbs sampler of 10,000 iterations after a 15,000 burn-in and thinning every five iterations. Posterior median estimates were derived from the MCMC output results. Flat, symmetric Dirichlet priors were fit for the probability of class assignment, π, and the food item probability of consumption given assignment to specific latent class, $\theta_{j\cdot |k}$ in accordance with the simulation study, with hyperparameters $\alpha =\frac{1}{K}$ and γ=1. A random permutation sampler was implemented to encourage mixing (Frühwirth-Schnatter, 2001). Dietary weights were calibrated and normalised for inclusion in analysis. Diagnostics and convergence of MCMC output were evaluated through visualisation of trace plots and the potential scale reduction factor.

All data included for this study and code to reproduce the derived dataset and perform subsequent analyses are made available on the author’s GitHub repository: http://www.github.com/bjks10/NHANES_wtofm. Dietary data were originally obtained from the NHANES website (https://wwwn.cdc.gov/nchs/nhanes) and processed in SAS 9.4. Statistical analysis, diagnostics, and figures were performed in MATLAB 2021a. Post-hoc analysis and table summaries were generated in R version 4.0.2.

### 4.2 wtOLCM results

**Figure 3.**
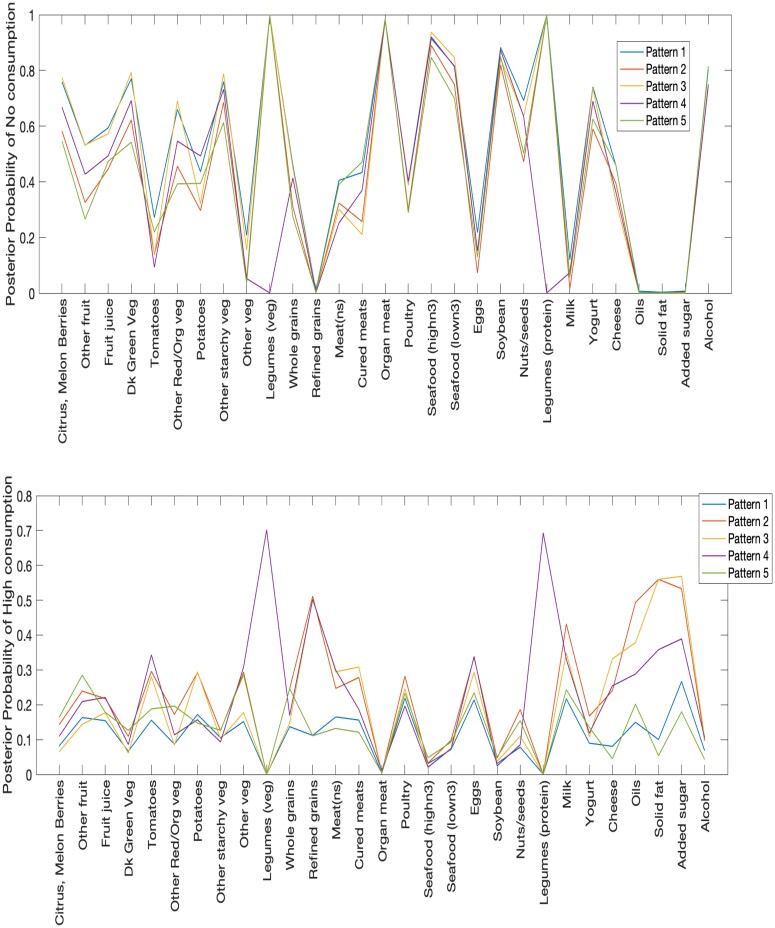
Low-income adult population: (top) Posterior mean probability of no consumption of a given food item given membership in a specified pattern; and (bottom) Posterior mean probability of high consumption of a given food item given membership in a specified pattern.

The weighted overfitted latent class model identified five nonempty clusters in the low-income adult population. Diagnostics and convergence were confirmed through trace plots and potential scale reduction factors of all parameters ranging from 0.999 to 1.014. Figure 3 illustrates the posterior mean estimates of the probability of no consumption or high consumption given membership to a given dietary pattern. From this figure, we can see which foods were strongly favoured to be consumed for various patterns. The very low probabilities of no consumption across all patterns for refined grains, oils, solid fats, and added sugar imply a general nonzero consumption by all low-income adults.

Foods such as poultry, seafood, eggs, soybean, and alcohol shared similar consumption behaviours across all diet patterns, but other foods differed by pattern. For example, Patterns 1 and 5 had the lowest probabilities for consumption of cheese, oils, added sugars, and fats at the high consumption level. Patterns 2 and 3 had the highest probabilities for consumption of refined grains, potatoes, cured meats, oils, solid fat, and added sugar at the high consumption level. Lastly, Pattern 4 distinctly had the highest probability of legumes being consumed at the high consumption level. Dietary Pattern 1, followed by Pattern 3, had the highest probabilities of no consumption of most fruits and vegetables.

**Figure 4.**
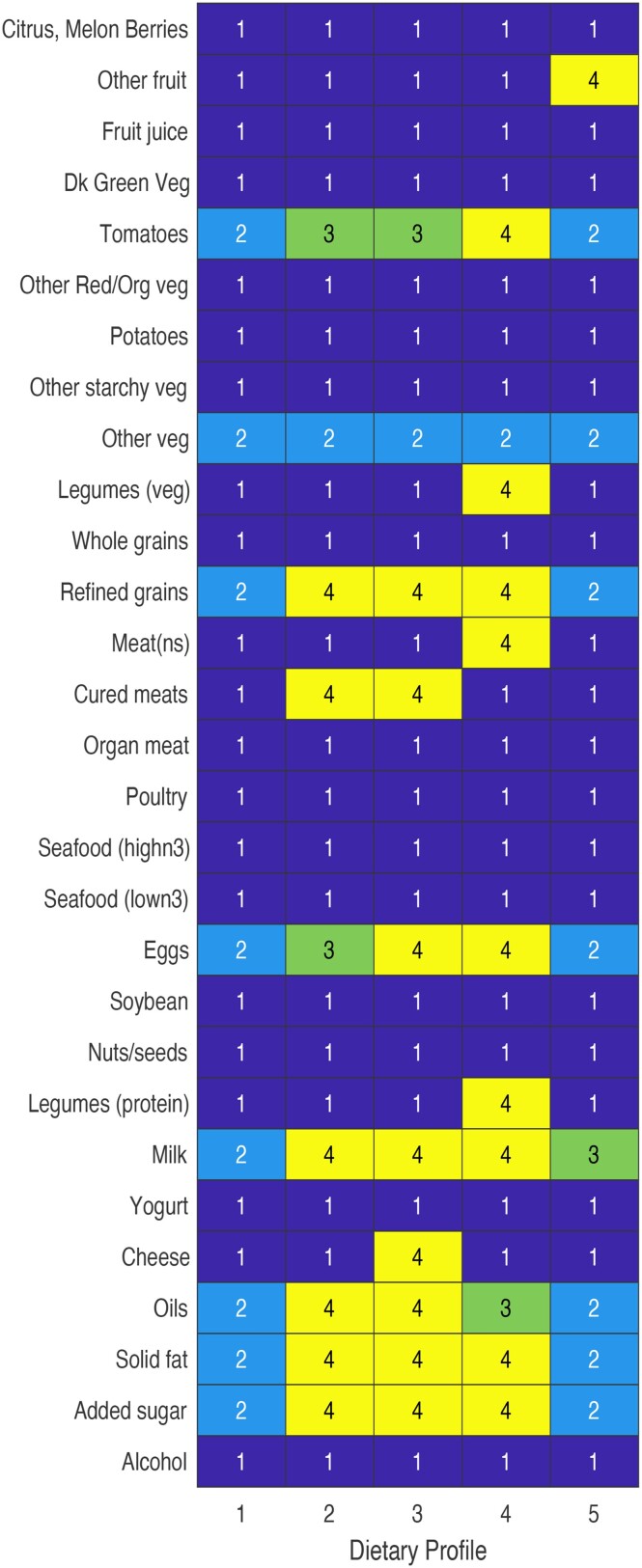
Posterior mode of consumption pattern of dietary patterns for non-incarcerated adults living at or below the 130% poverty level. Numbers represent levels of consumption: 1=None, 2=Low, 3=Medium, 4=High.

Comparing more closely the posterior modes of consumption for each dietary pattern, we note that 15 foods shared a mode of non-consumption (i.e. consumption value of 1) across the five dietary patterns (Figure 4): citrus/melon/berries, fruit juice, dark green vegetables, other red/orange vegetables, potatoes, other starchy vegetables, whole grains, organ meat, poultry, seafood (high-n3), seafood (low-n3), soybean, nuts/seeds, yogurt, and alcohol. Pattern 1 showed strong similarities with Pattern 5. However, Pattern 5 had comparatively higher levels of consumption of other fruit and milk. Patterns 2 and 3 also shared similar consumption of foods, with differences noted in the higher level of consumption for eggs and cheese in Pattern 3. As previously noted, Pattern 4 was the most distinguishable among the five patterns, with a high level of consumption favoured in tomatoes, legumes (veg and protein), and non-specified meat.

Table 1 provides a summary of the demographics for participants assigned to each dietary pattern. Among the low-income adult population, participants assigned to Pattern 5 had the highest average HEI-2015 score (57.4±0.6). This pattern favoured a high consumption of other fruit, but a low consumption of refined grains and no consumption of meats. Pattern 3 had the lowest average HEI-2015 score (41.1±0.3). This pattern favoured a high consumption of refined grains, cured meats, eggs, cheese, fats, oils, and sugars. Demographically, we observe that those in Pattern 5 were predominantly male adults, whereas those assigned to Pattern 3 were predominantly female adults. While non-Hispanic White participants held the majority of all patterns in our model, Pattern 4, which uniquely favoured a high consumption of legumes, was the only pattern where minority participants had a higher representation.

**Table 1.** Demographic distribution of low-income dietary patterns

	Pattern 1		Pattern 2		Pattern 3		Pattern 4		Pattern 5	
	Mean	SE	Mean	SE	Mean	SE	Mean	SE	Mean	SE
Overall	21.7	0.6	11.7	0.6	22.0	0.7	29.3	1.0	15.4	0.9
Race/Ethnicity										
Mexican	10.2	1.7	12.2	1.6	11.0	1.5	26.4	2.9	10.8	1.5
Other Hispanic	8.4	1.2	7.5	1.2	5.2	0.9	15.7	1.8	7.9	1.0
Non-Hispanic White	50.5	2.9	54.3	3.4	55.1	2.7	37.0	2.8	47.9	3.2
Non-Hispanic Black	21.2	2.1	18.2	2.1	22.1	2.6	11.1	1.2	16.8	1.8
Non-Hispanic Asian	5.4	0.9	4.5	0.8	1.2	0.2	5.8	0.9	12.5	1.8
Mixed/Other	4.4	1.0	3.3	0.7	5.5	0.7	4.0	0.8	4.2	0.9
Gender										
Male	63.2	1.5	47.2	2.5	38.6	1.5	53.6	1.5	72.2	2.1
Female	36.8	1.5	52.8	2.5	61.4	1.5	46.4	1.5	27.7	2.1
Age Group										
20–34 years	33.1	1.9	38.2	2.8	46.6	2.5.	35.2	2.1	22.8	2.0
35–49 years	24.9	1.5	20.5	1.6.	25.6	1.5	26.6	1.4	20.0	1.5
50–64 years	26.1	1.4	22.5	2.0	18.2	1.6	25.3	1.7	27.3	2.1
65+ years	15.9	1.1	18.8	2.1	9.6	1.0	12.9	1.0	30.0	2.1
HEI 2015 Score	45.8	0.5	50.3	0.6	41.1	0.3	53.2	0.5	57.4	0.6
Framingham 10-year Risk	7.7	0.3	7.6	0.4	7.1	0.4	7.4	0.3	9.3	0.5
CVD Risk factors										
Hypertension	34.9	2.1	30.9	3.5	30.9	2.0	30.2	1.7	36.4	2.8
Hypercholesteremia	76.7	1.9	67.9	4.2	69.4	1.9	75.5	1.9	68.1	2.5
Obesity	45.7	2.6	42.2	3.8	41.4	2.5	40.1	1.9	38.0	2.7
Diabetes	12.8	1.0	10.0	1.6	8.2	1.2	9.1	1.1	14.2	1.6
Smoker	34.5	3.1	18.6	2.5	34.9	3.0	19.2	2.1	11.9	1.8

### 4.3 Comparison to unweighted model

Ignoring the weights in the survey data generates different pattern results and prevalence. The cohort-specific model generated six clusters, ranging in size of 8.5% (unweighted Pattern 3) to 30% (unweighted Pattern 5). Consistent with what we saw in the simulation case, the OLCM of the cohort sample had similar patterns with the addition of a new pattern that looks like a mix of two separated patterns in the weighted model. A comparison of the consumption modes to describe each diet pattern for each model is provided in online supplementary material Figure 2. About 97% of the cohort participants that were assigned to the largest pattern in the unweighted model also contributed to the largest pattern of the weighted model. Yet, the consumption modes describing the respective patterns differed for four foods: non-specified meats, oils, solid fat, milk. For example, in the unweighted model, there was a 44% probability of not consuming non-specified meat for unweighted Pattern 5 compared to 25% for weighted Pattern 4. The smallest pattern prevalence identified in the unweighted model (Pattern 3) differed in consumption of two foods from the weighted model (Pattern 2): cured meats and tomatoes. These differences further highlight the consequence of applying survey data without the weights to appropriately estimate the true population, as opposed to the study cohort.

## 5 Discussion

The wtOLCM for survey data, proposed in this paper, is an extension of the standard latent class model and integrates a Bayesian nonparametric survey-weighted approach to account for sampling variability in its parameter estimation. Our simulation study compared our proposed model with other standard approaches used for survey data. The results showed that wtOLCM had an improved estimation of the true population prevalence as well as pattern identification, particularly as more heterogeneity is introduced. We applied our model to dietary survey data collected in the 2011–2018 National Health and Nutrition Examination Surveys in order to better examine the dietary patterns of US adults living at or below the 130% poverty income level. Our model identified five dietary patterns in this sampled subset. Application of our model to this target population allowed us to leverage survey weights to obtain representative estimates from a smaller, often underrepresented and understudied, subset of the surveyed participants. Ignoring the weights provided by the survey would have biased our results and led to misleading inference of this low-income adult population.

This method builds its strength on its generalisability and use in nationally representative dietary surveys, yet recognises the concerns of overgeneralisation. Dominating demographics can still influence pattern distribution in a given population. Non-Hispanic White participants have historically dominated surveys and studies that examine diet-disease relationships (Fahlman et al., 2010; Ohlhorst et al., 2013). This underrepresentation of minority subgroups makes it difficult to identify a uniquely separate cluster under the global clustering assumption, if the derived pattern is not shared among all individuals. An overrepresentation of this subgroup can mask accurate pattern identification for racial/ethnic minorities who may be at greatest risk of chronic disease. This is exemplified in our model through Pattern 4, which had the most distinguishable dietary consumption pattern. Compared to the other five patterns, this contained the smallest proportion of non-Hispanic White adults, but still the largest relative proportion among the other racial/ethnic subgroups. If certain subgroups are important to understand nutrition disparities, those subgroups should be studied in a separate analysis or a more advanced method that is able to jointly account for subgroup differences should be implemented. To our knowledge, few advanced methods have been used to better examine subpopulation behaviour differences, but incorporating the complex survey design directly into model estimation has not yet been fully explored (De Vito et al., 2019; Stephenson & Willett, 2023).

While the utility of this model has effectively demonstrated its use in diet survey data, we must also acknowledge the limitations with the model. Latent class or mixture models are prone to sampling bias. Different sampled populations could yield different model results and characterised dietary patterns. In survey studies, this can impose even wider variation given that the model assumes the sampled population accurately reflects those comprising the non-sampled target population. New methods are emerging that have tried to mitigate the sampling bias limitation of non-sampled units (S. Chen et al., 2022; Y. Chen et al., 2020; J. K. Kim et al., 2021). However, these methods require auxiliary information to be known about the non-sampled population, which is a challenge concerning the unique variation present in dietary intake. This paper did not report estimates of variance and uncertainty for the wtOLCM. These estimates are expected to exhibit slightly less than nominal coverage given the sampling variation. Though methods have been proposed to address this issue (León-Novelo & Savitsky, 2019; Williams & Savitsky, 2021), incorporation of these methods into a model-based clustering framework, such as the wtOLCM, remains an area of active research and has yet to be explored through the scientific lens of dietary pattern identification and characterisation.

Another limitation with the use of dietary intake data is its reliance on self-reporting. Several nutrition studies have found that prudent foods like vegetables and fruits are often overreported and less prudent foods like fats and oils are frequently underreported (Amanatidis et al., 2001; Haraldsdóttir, 1993). These tendencies to misreport have been associated with demographics such as BMI, age, sex, socioeconomic status, as well as other psychosocial and cognitive factors (Klesges et al., 1995; Poslusna et al., 2009; Tooze et al., 2004). Methods such as doubly labelled water and biomarkers for select nutrients are available to validate dietary assessment tools, but these instruments are beyond the scope of tools utilised in the National Health and Nutrition Examination Survey. In spite of this limitation, the misreporting rate remains relatively low and the instruments can still be deemed relatively reliable (Tooze et al., 2004; Yuan et al., 2017). Another limitation of dietary recalls is the inability to capture day-to-day variation. As a result, these dietary patterns are based on one or two days of dietary records, which may or may not reflect participants’ regular dietary behaviours. Alternative dietary assessments, such as food frequency questionnaires and 7-day daily diet records, are available to capture more episodic and rarely consumed foods. However, more detailed assessments are often costly and seldom widely available in large population-based surveys. Future research can explore ways to integrate these tools, when available, to quantify the unknown variation and uncertainty that comes from misreporting in dietary assessments.

The method proposed in this study relies on complete dietary intake data. Data imputation strategies are available prior to model implementation to resolve any food items that may be missing. This was of minimal concern for us since NHANES did not have any missing item response data within a survey. Some participants were missing second dietary recalls, due to loss of follow-up. However, sensitivity analysis did not reveal any significant changes to results that had only one available recall compared to two. Our results also pooled data across four NHANES reporting cycles, starting in 2011. This was done in an effort to allow greater precision and representation of smaller-sized minority groups (e.g. Other Hispanic and Non-Hispanic Asian Americans). As a repeated cross-sectional study, each survey cycle had a unique set of individuals. While temporal variation in these diets may be a concern, prior studies have found very little year-to-year variation in overall dietary patterns during this time frame, which was confirmed through sensitivity analysis (Ansu Baidoo et al., 2022; Nagao-Sato & Reicks, 2022; Stephenson & Willett, 2023). However, with larger time frame variation, further research is needed to explore methodological extensions to account for how these patterns may change over time.

The clustering approach applied in this paper, as well as more traditionally used cluster and factor analysis, are all generated independent of any health outcome. Yet, when looking at exposures from a multi-dimensional perspective, these exposures may be driven by an underlying health outcome, in which case a more supervised approach may yield more useful information to understand how the combination of these exposures (e.g. dietary habits) can drive a known outcome (e.g. cardiometabolic health). Further research is needed to develop supervised clustering methods that address the issue of confounding overgeneralisations and are applicable in population surveys with complex survey designs.

## Supplementary Material

qnad135_Supplementary_Data

## Data Availability

Software in the form of MATLAB code, together with a sample input data set and complete documentation to replicate analysis is available in the GitHub repository https://github.com/bjks10/NHANES_wtofm.
