# Supplementary material for: Identifying dietary consumption patterns from survey data: a Bayesian nonparametric latent class model
Source: J R Stat Soc Ser A Stat Soc. 2023 Dec 12;187(2):496–512. doi: 10.1093/jrsssa/qnad135 (PMC11009925; doi:10.1093/jrsssa/qnad135)
Supplement: qnad135_Supplementary_Data [file qnad135_supplementary_data.pdf]

SUPPORTING WEB MATERIALS FOR IDENTIFYING DIETARY CONSUMPTION PATTERNS FROM SURVEY DATA: A BAYESIAN NONPARAMETRIC LATENT CLASS MODEL

STEPHENSON ET AL.

Contents

1. Weighted Finite Population Bayesian Bootstrap method 1
References 2
2. Posterior computation of weighted overfitted latent class model 2
3. Supplementary Tables 3
4. Supplementary Figures 3

1. Weighted Finite Population Bayesian Bootstrap method

In the weighted finite population Bayesian bootstrap (WFPBB) method, the population from which the survey data arise is assumed to be finite. A pseudo-representative sample (PRS) approximates a simple random sample from the finite population. To generate a PRS, an "unweighted" synthetic population is first created using a weighted multiple imputation approach, where non-sampled units in the finite population are viewed as missing data and are imputed by sampling from the posterior predictive distribution given the sampled data and the survey weights. Imputation proceeds via the WFPBB method (Dong et al., 2014; Cohen, 1997), which extends the Bayesian bootstrap (Rubin, 1981) and its weighted version (Lo, 1993) to also account for a finite population. Dong et al. (2014) show that sampling from the posterior predictive distribution of the non-sampled units given the sampled data and survey weights is equivalent to sampling from a weighted Pólya urn scheme. This allows for simple implementation of the procedure to generate the synthetic population. A total of 200 synthetic populations are generated, and the corresponding 200 PRS's are formed by taking a simple random sample without replacement from a synthetic population.

Posterior inference is obtained by running the unweighted overfitted latent class model on each PRS as if it were the actual sampled data, and then combining results across the samples. Parameter point (and variance) estimates are obtained by taking the mean of the posterior median (and variance) estimates across all PRS's. One notable limitation of this procedure is that it does not allow for classification of individuals into clusters. Incorporation of survey weights into Bayesian clustering methods that allow for classification within a multiple imputation framework is a potentially promising direction for future research.

References

[1] Cohen, M. P. (1997) The bayesian bootstrap and multiple imputation for unequal probability sample designs. In *Proceedings of the Survey Research Methods Section, American Statistical Association*, 635–638.

[2] Dong, Q., Elliott, M. R. and Raghunathan, T. E. (2014) A nonparametric method to generate synthetic populations to adjust for complex sampling design features. *Survey methodology*, **40**, 29.

[3] Lo, A. Y. (1993) A bayesian method for weighted sampling. *The Annals of Statistics*, **21**, 2138–2148.

[4] Rubin, D. B. (1981) The bayesian bootstrap. *The annals of statistics*, 130–134.

2. Posterior computation of weighted overfitted latent class model

(1) Update z_i for $i \in (1, \ldots, n)$:

$$Pr(z_i = k) = \frac{\pi_h \prod_{j=1}^{p} \prod_{c=1}^{d_j} \theta_{jc|h}^{1(y_{ij}=c)}}{\sum_{k=1}^{K} \pi_k \prod_{j=1}^{p} \prod_{c=1}^{d_j} \theta_{jc|k}^{1(y_{ij}=c)}}$$

(2) Update $\pi = (\pi_1, \ldots, \pi_K)$:

$$\pi_{1:K} \sim \text{Dir}\left(\alpha + \sum_{i=1}^{n} \mathbf{1}(z_i = 1) \times \frac{w_i}{\kappa}, \ldots, \alpha + \sum_{i=1}^{n} \mathbf{1}(z_i = K) \times \frac{w_i}{\kappa}\right),$$

(3) Update $\theta_{j\cdot|h} = (\theta_{j1|k}, \ldots \theta_{jd|k})$ for all $j \in (1, \ldots, p), k \in (1, \ldots, K)$:

$$\theta_{j\cdot|k} \sim \text{Dir}_{d_j}\left(\eta + \sum_{i:z_i=k} \mathbf{1}(y_{ij} = c|z_i = k) \times \frac{w_i}{\kappa}\right)$$

Table 1. Distribution of patterns across simulated population containing four subpopulations and three uniquely distinct patterns

	**Pattern 1**	**Pattern 2**	**Pattern 3**	**Total**
Subpopulation 1	25	900	75	1000
Subpopulation 2	0	175	1825	2000
Subpopulation 3	25	425	1050	1500
Subpopulation 4	450	0	50	500
Total	500	1500	3000	5000

Table 2. NHANES 2011-2018 adult participant demographics

	Overall			Low-income		
Demographics	N	%	(SE)	N	%	(SE)
**Race/Ethnicity**						
Mexican	2632	9.2	(1.0)	1353	15.4	(1.7)
Other Hispanic	2026	6.3	(0.6)	950	9.7	(1.0)
Non-Hispanic White	7480	64.0	(1.7)	2390	47.6	(2.3)
Non-Hispanic Black	4471	11.3	(1.0)	1963	17.4	(1.5)
Non-Hispanic Asian	2282	5.8	(0.5)	609	5.6	(0.7)
Mixed/Other	716	3.4	(0.3)	296	4.3	(0.5)
**Gender**						
Male	10077	48.8	(0.5)	3517	45.5	(0.8)
Female	9530	51.2	(0.5)	4044	54.5	(0.8)
**Age**						
20-34 Years	4991	28.9	(0.8)	2129	35.7	(1.5)
35-49 Years	4801	25.5	(0.7)	1767	24.3	(0.9)
50-64 Years	5246	26.9	(0.6)	1976	23.9	(0.9)
65+ Years	4569	18.8	(0.6)	1689	16.1	(0.8)
AHEI2015 Score	14865	51.6	(0.3)	5823	49.2	(0.3)
Framingham 10YR Score	18226	8.1	(0.1)	6904	7.7	(0.2)
**CVD Risk factors**						
Hypertension	8260	32.6	0.9	2446	32.3	1.0
Obesity	8198	39.0	0.9	2589	41.4	1.2
Diabetes	2010	9.3	0.4	643	10.6	0.6
High Cholesterol	9906	75.1	0.8	2874	72.4	1.0
Smoker	3487	15.1	0.7	1605	24.4	1.4

3. Supplementary Tables

4. Supplementary Figures

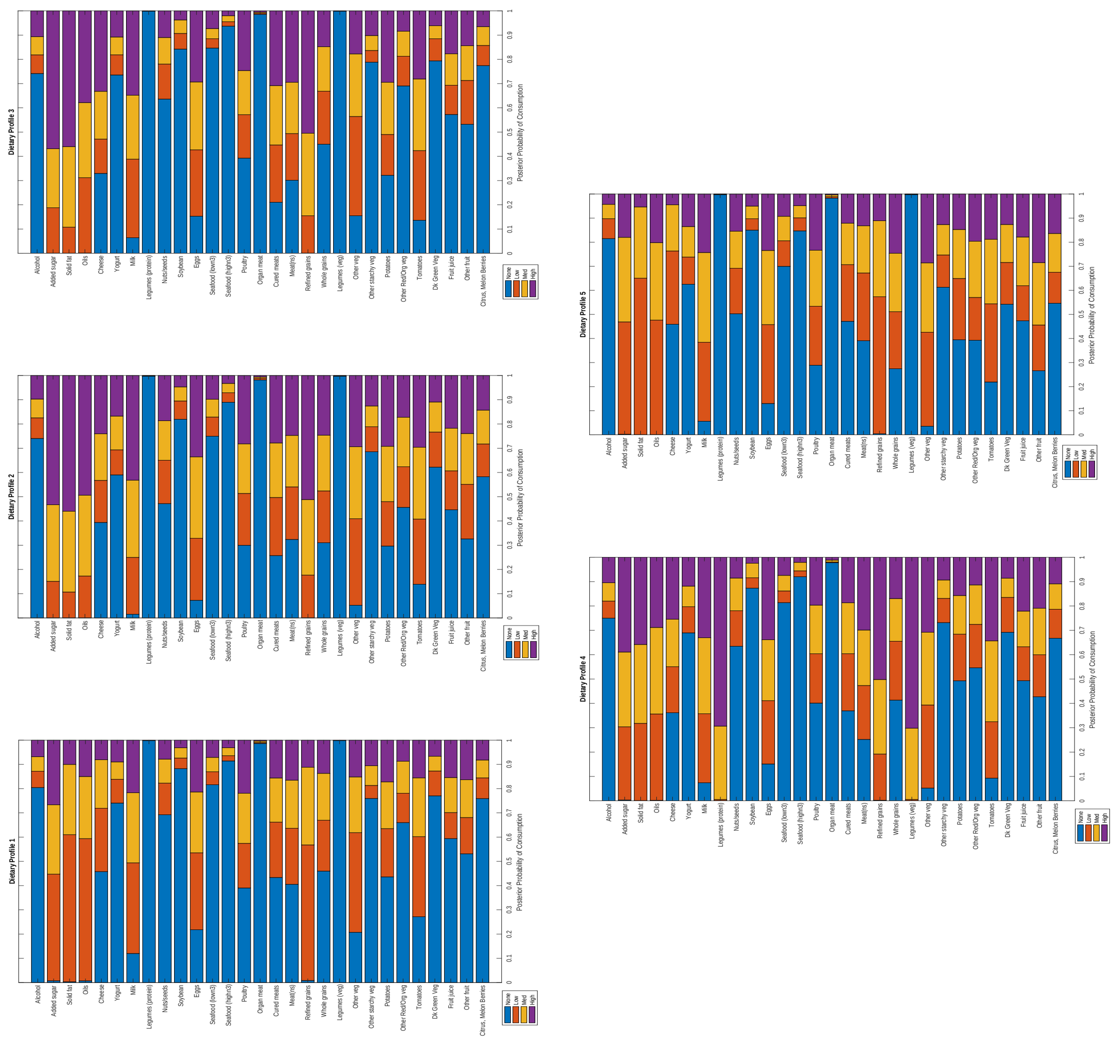

FIGURE 1. Distribution of dietary consumption for each dietary pattern derived from NHANES data on Low-income adult population

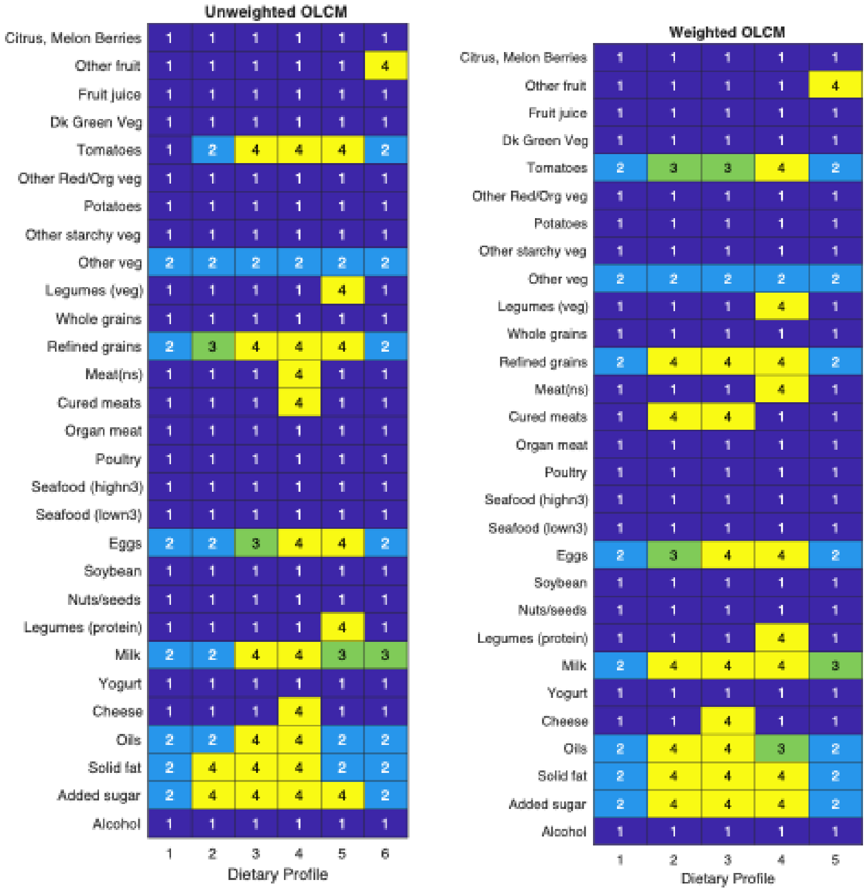

FIGURE 2. Posterior mode of consumption pattern of dietary patterns for non-incarcerated adults living at or below the 130% poverty level. Left=unweighted model with (π_{OLCM} = $0.18, 0.13, 0.09, 0.16, 0.30, 0.16$). Right = weighted model with (π_{wtOLCM} = $0.22, 0.12, 0.22, 0.29, 0.15$). Numbers represent levels of consumption: 1= None, 2=Low, 3=Medium, 4=High.
